# On the Importance of Collaboration

**DOI:** 10.19102/icrm.2018.090104

**Published:** 2018-01-15

**Authors:** Andy C. Kiser, John Mark Williams


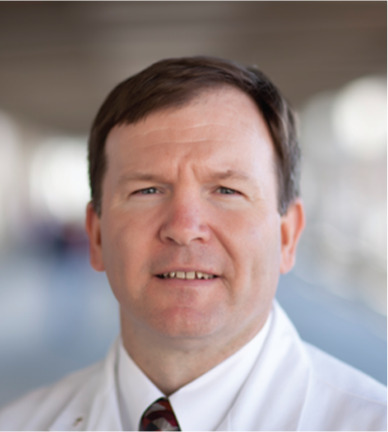


In the clinic, the presence of the transcatheter aortic valve replacement heart team highlights and clearly demonstrates the success of medical collaboration among those with different clinical backgrounds. Similarly, a collaborative approach to cardiac rhythm management has gained momentum among physicians with the expansion of hybrid atrial fibrillation procedures. Together, surgeons and electrophysiologists work side-by-side, combining their expertise for the good of the patient while simultaneously learning from each other so as to further enhance future treatment efforts. This opportunity to share knowledge, techniques, and technology has led to the present introduction of a new section, Surgical Rhythm Management, in *The Journal of Innovations in Cardiac Rhythm Management*.

At this time, the editors of *The Journal of Innovations in Cardiac Rhythm Management* and I invite our surgical colleagues to submit manuscripts on surgical ablation, perioperative rhythm management, lead and device extraction, and other topics pertaining to cardiac rhythm management. We also encourage contributions about communication and multidisciplinary team-building efforts between electrophysiologists and surgeons. The section on Surgical Rhythm Management will focus on mutually interesting topics and innovative ideas designed to enhance collaboration across our specialties. It will also provide an avenue for surgeons to share their experiences and expertise with the electrophysiology community.

In the coming months, articles exemplifying these objectives will introduce this journal’s audience to surgical leaders who have demonstrated a passion for rhythm management. I invite all those who are interested to freely suggest contributions and contributors as we build an outlet for scholarly collaboration.

With warmest regards,


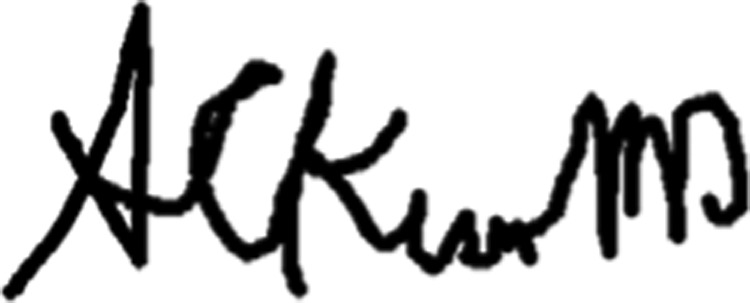


Andy C. Kiser, md, facs, facc, fccp

kisera16@ecu.edu

John Mark Williams, md, Distinguished Professor in Cardiac Surgery

Chief, Division of Cardiac Surgery

Department of Cardiovascular Sciences

East Carolina Heart Institute at East Carolina University

Greenville, NC 27834, USA

